# Rationale of the BREAst cancer e-healTH [BREATH] multicentre randomised controlled trial: An Internet-based self-management intervention to foster adjustment after curative breast cancer by decreasing distress and increasing empowerment

**DOI:** 10.1186/1471-2407-12-394

**Published:** 2012-09-07

**Authors:** Sanne W van den Berg, Marieke F M Gielissen, Petronella B Ottevanger, Judith B Prins

**Affiliations:** 1 Department of Medical Psychology, Radboud University Medical Centre, Nijmegen, the Netherlands; 2 Department of Medical Oncology, Radboud University Medical Centre, Nijmegen, the Netherlands

**Keywords:** Breast cancer, Internet, Self-management, Intervention, Adjustment, Empowerment, eHealth, RCT

## Abstract

**Background:**

After completion of curative breast cancer treatment, patients go through a transition from patient to survivor. During this re-entry phase, patients are faced with a broad range of re-entry topics, concerning physical and emotional recovery, returning to work and fear of recurrence. Standard and easy-accessible care to facilitate this transition is lacking. In order to facilitate adjustment for all breast cancer patients after primary treatment, the BREATH intervention is aimed at 1) decreasing psychological distress, and 2) increasing empowerment, defined as patients’ intra- and interpersonal strengths.

**Methods/design:**

The non-guided Internet-based self-management intervention is based on cognitive behavioural therapy techniques and covers four phases of recovery after breast cancer (Looking back; Emotional processing; Strengthening; Looking ahead). Each phase of the fully automated intervention has a fixed structure that targets consecutively psychoeducation, problems in everyday life, social environment, and empowerment. Working ingredients include Information (25 scripts), Assignment (48 tasks), Assessment (10 tests) and Video (39 clips extracted from recorded interviews). A non-blinded, multicentre randomised controlled, parallel-group, superiority trial will be conducted to evaluate the effectiveness of the BREATH intervention. In six hospitals in the Netherlands, a consecutive sample of 170 will be recruited of women who completed primary curative treatment for breast cancer within 4 months. Participants will be randomly allocated to receive either usual care or usual care plus access to the online BREATH intervention (1:1). Changes in self-report questionnaires from baseline to 4 (post-intervention), 6 and 10 months will be measured.

**Discussion:**

The BREATH intervention provides a psychological self-management approach to the disease management of breast cancer survivors. Innovative is the use of patients’ own strengths as an explicit intervention target, which is hypothesized to serve as a buffer to prevent psychological distress in long-term survivorship. In case of proven (cost) effectiveness, the BREATH intervention can serve as a low-cost and easy-accessible intervention to facilitate emotional, physical and social recovery of all breast cancer survivors.

**Trial registration:**

This study is registered at the Netherlands Trial Register (NTR2935)

## Background

A growing number of women is living with and beyond breast cancer. The incidence in the Netherlands is expected to grow with 27% between 2010 and 2020 [1], and reflects the increasing breast cancer incidence of women worldwide [2,3]. At the same time, the national biennial mammographic screening for all women aged 50–75 in the Netherlands has resulted in a decrease in mortality in women with breast cancer [4]. As a result, nowadays the majority of women undergo curative treatment and continue to live their life with breast cancer as a chronic illness. This view of breast cancer as a chronic illness has implications for follow-up or survivorship care [5], and poses new challenges to deliver care to the increasing population of breast cancer survivors (BCS).

### The re-entry phase

After the end of primary curative breast cancer treatment, patients go through the transition from ‘patient’ to ‘survivor’ [6,7]. This transition or re-entry phase [8] is characterized by multiple adaptive tasks on emotional, physical and social domain and sets stage for adaptive long-term survivorship [9]. Topics encountered during the re-entry phase are in principle universal for all BCS and include among others: physical recovery, emotional processing, fear of recurrence, decreasing social support (losing the “safety net” of treatment), resuming professional activities, but also positive life changes (e.g. valuing life more) [6,7,10-12]. Although the majority of BCS eventually adjusts well [13,14], there is a high information need concerning these topics [15]. However, despite the high information need and universality of re-entry topics, standardized and easy-accessible care to facilitate the transition towards breast cancer survivorship is lacking.

### Interventions during re-entry phase

Research on the effectiveness of psychosocial interventions to improve psychological well-being after completion of curative breast cancer treatment is still scarce and inconclusive [10,16]. With regard to the type of intervention it is suggested that interventions based on cognitive behavioural therapy (CBT) produce larger effect sizes than interventions lacking CBT components [17], and can be effective in reducing distress [18] and improving quality of life in BCS [16]. Also, a central role in survivorship care has been proposed for self-management [5], which is defined as “the individual’s ability to manage the symptoms, treatment, physical and psychosocial consequences and life style changes inherent in living with a chronic condition” [19]. First evidence suggests that self-management interventions in the re-entry phase can facilitate the transition into breast cancer survivorship. For example, pilot testing of a self-management intervention with face-to-face and telephone contact (the Taking Charge intervention [20]) showed beneficial effects on dealing with post-treatment concerns. The Moving Beyond Cancer randomised controlled trial [21] demonstrated beneficial effects of psychoeducational print material and peer modeling videos for BCS on regaining energy in the re-entry phase.

### Internet interventions

Evidence is growing that Internet interventions can improve psychological well-being in cancer patients [22]. Compared to other methods of delivery, the Internet provides an easily accessible opportunity to reach the large group of cancer survivors. Also, Internet interventions can contain more channels of media to tailor information, and can provide more anonymity compared to face-to-face interventions. Other advantages include avoiding waiting lists, providing consistency of care, and a 24-hour availability [23]. Pilot evidence suggests that an Internet intervention specially designed for posttreatment survivors (Project Onward [24]) can have high utilization rates and reduce depressive symptoms. However, despite the large number of women turning to the Internet for breast cancer-related information [25-27], evidence-based Internet interventions specifically designed for BCS in the re-entry phase are lacking.

### BREast cancer e-healTH [BREATH]

The BREAst cancer e-healTH [BREATH] intervention (‘Catching your breath after breast cancer’) is a non-guided Internet-based self-management website for BCS aiming to foster adjustment after completion of primary curative treatment. Self-management interventions are multi-component and “usually designed to increase the repertoire of participants’ self-management skills within the realities of living with a chronic condition” [28]. The BREATH intervention is a self-management program based on CBT containing components such as psychoeducation, cognitive reframing, goal planning and process evaluation. By using the intervention, BCS will learn how to use these CBT techniques as self-management skills in their daily lives. The BREATH intervention has a fixed content and structure, because it is assumed that “effective self-managers will feel confident in selecting the techniques(s) that they believe will meet their specific needs at a given point of time and in a given environment or situation” [28]. Peer-support is not included in the BREATH intervention, since in the Netherlands support-groups are already widely accessible on the Internet. Also, scientifically the use of Internet-based support groups in cancer patients still needs to confirm long-lasting psychological effects [29]. The BREATH intervention is designed to facilitate and promote adjustment for all BCS, both distressed and non-distressed. In concordance with the stress-coping model of Leventhal (1984), emotional well-being after cancer is defined as the balance between stress and resources [30]. Therefore, in order to target these two aspects of emotional well-being, the aim of the BREATH intervention is twofold: decreasing psychological distress and increasing psychological empowerment, which reflects the individual outcome measure of a patients’ intrapersonal and interpersonal strengths [31].

### Hypotheses of the BREATH trial

The primary objective of this randomised controlled trial (RCT) is whether the BREATH intervention is effective compared to usual care in fostering adjustment after curative breast cancer treatment by reducing psychological distress and improving empowerment in BCS. Because BSC with and without elevated levels of psychological distress are included it is hypothesized that through using the BREATH intervention:

1) *distressed* BCS will experience a decrease in psychological distress,

2) *non-distressed* BCS will maintain a low level of distress, and/or

3) both *distressed* and *non-distressed* BCS will increase in empowerment.

## Methods/design

In this article, the BREATH study design and intervention will be reported in concordance with the guidelines of reporting Internet intervention research [22] and the CONSORT 2010 statement for reporting parallel group randomised trials [32], and eHealth interventions [33].

### Study design

**Figure 1:**
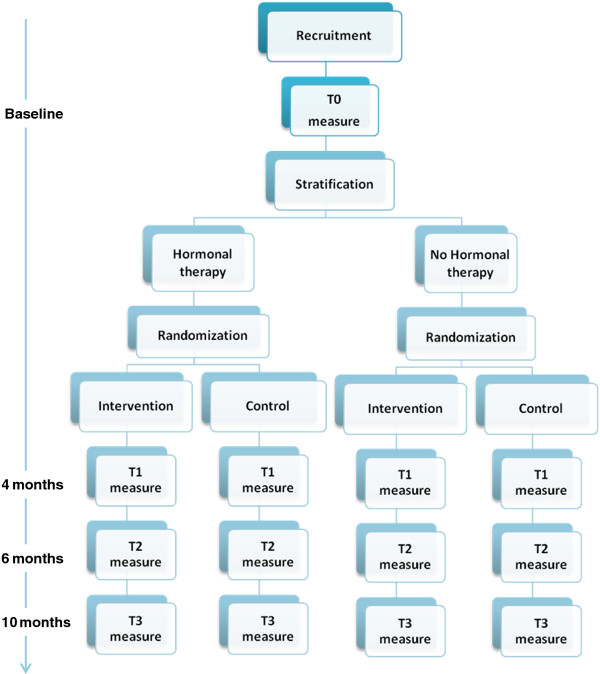
Overall study design of the BREATH study.

This study is designed as a non-blinded, multicentre randomised controlled, parallel-group trial evaluating the superiority of the BREATH intervention compared to usual care after primary curative breast cancer treatment. A consecutive sample of 170 BCS from 6 hospitals will be evaluated in this RCT. Baseline measure (T0) and randomization take place 3 months after completion of primary curative breast cancer treatment. This starting point was chosen because this is in accordance with the standard first follow-up visit after completion of primary treatment as described in the Dutch national breast cancer guideline. Moreover, these 3 months allow for the natural recovery of emotional well-being and adjustment to take place. After completion of baseline measure, participants will be randomised to either intervention or control group. Follow-up measures are respectively 4 months (T1; post-intervention), 6 months (T2), and 10 months (T3) after baseline. The overall study design is captured in Figure 1.

### Participant eligibility

Inclusion criteria are: Women with a histologically proven malignancy of the breast; breast cancer is treated with curative intent surgery and adjuvant chemotherapy and/or radiotherapy; last chemo- or radiotherapy is received ≥2 and ≤4 months; direct access to a computer with Internet connection; basic Internet skills (e.g. in possession of email address); and a good command of the Dutch language. Exclusion criteria are: Men; breast cancer treated only with surgery; metastatic breast carcinoma; previous malignancy except adequately treated cervix carcinoma in situ and treated basal cell carcinoma of the skin; current treatment in psychiatric outpatient clinic.

### Recruitment settings and procedure

Participants will be recruited from the oncology and radiotherapy outpatient clinic of a university hospital (Radboud University Medical Centre, Nijmegen), and oncology outpatient clinics of five regional hospital sites (Rijnstate hospital, Arnhem and Zevenaar; Slingeland hospital, Doetinchem; hospital Gelderse Vallei, Ede; Canisius Wilhelmina hospital, Nijmegen; Jeroen Bosch hospital; Den Bosch). All hospitals involved in this multicentre RCT are situated in the southeastern part of the Netherlands.

Recruitment will take place at the end of curative treatment. During the last chemotherapy, or first follow-up visit (3 months after completion of primary treatment) eligible BCS are informed about the BREATH study by a member of their treatment team (oncologist, radiotherapist, or nurse). When BCS are interested in participating in the study, the researcher will have a onetime telephone contact (15–30 minutes) to provide additional information, address questions and second check of the eligibility criteria. Participants will complete informed consent during the next visit at their local hospital, or by mail (depending on the local informed consent procedure of the hospital setting).

### Randomization

The unit of randomization in this study is the individual breast cancer survivor. For each hospital setting, stratified randomization will be based on hormonal therapy. Adjuvant primary curative breast cancer treatment involves hormonal therapy in about 75% of all BCS. During hormonal therapy patients report mood swings and increased fatigue, which can be expected to influence the relationship between the intervention and the outcome variable. After stratification for the use of hormonal therapy, for each hospital a randomised block design will be used using variable block sizes of 4, 6 and 8 to ensure blinded allocation concealment. With an online computerized random number generator, participants will be randomly allocated to receive either usual care or usual care plus access to the online BREATH intervention with an allocation ratio of 1:1. Both the participants, their health care providers, and the researcher are blind to the allocation sequence, but the participant and the researcher are not blind for the randomization outcome.

### The BREATH intervention

The intervention is developed by the department of Medical Psychology of the Radboud University Medical Centre Nijmegen in the Netherlands, with technical assistance for ICT applications from Innovatie Psychologische en Psychiatrische Zorg (IPPZ), Utrecht in the Netherlands. The intellectual ownership of the intervention lies with the department of Medical Psychology. During the development of the intervention, patient participation was secured by (filmed) interviews with patients, content feedback by a multidisciplinary reading committee, including BCS and oncology professionals, and usability testing of the final website.

#### Intervention content and structure

**Figure 2:**
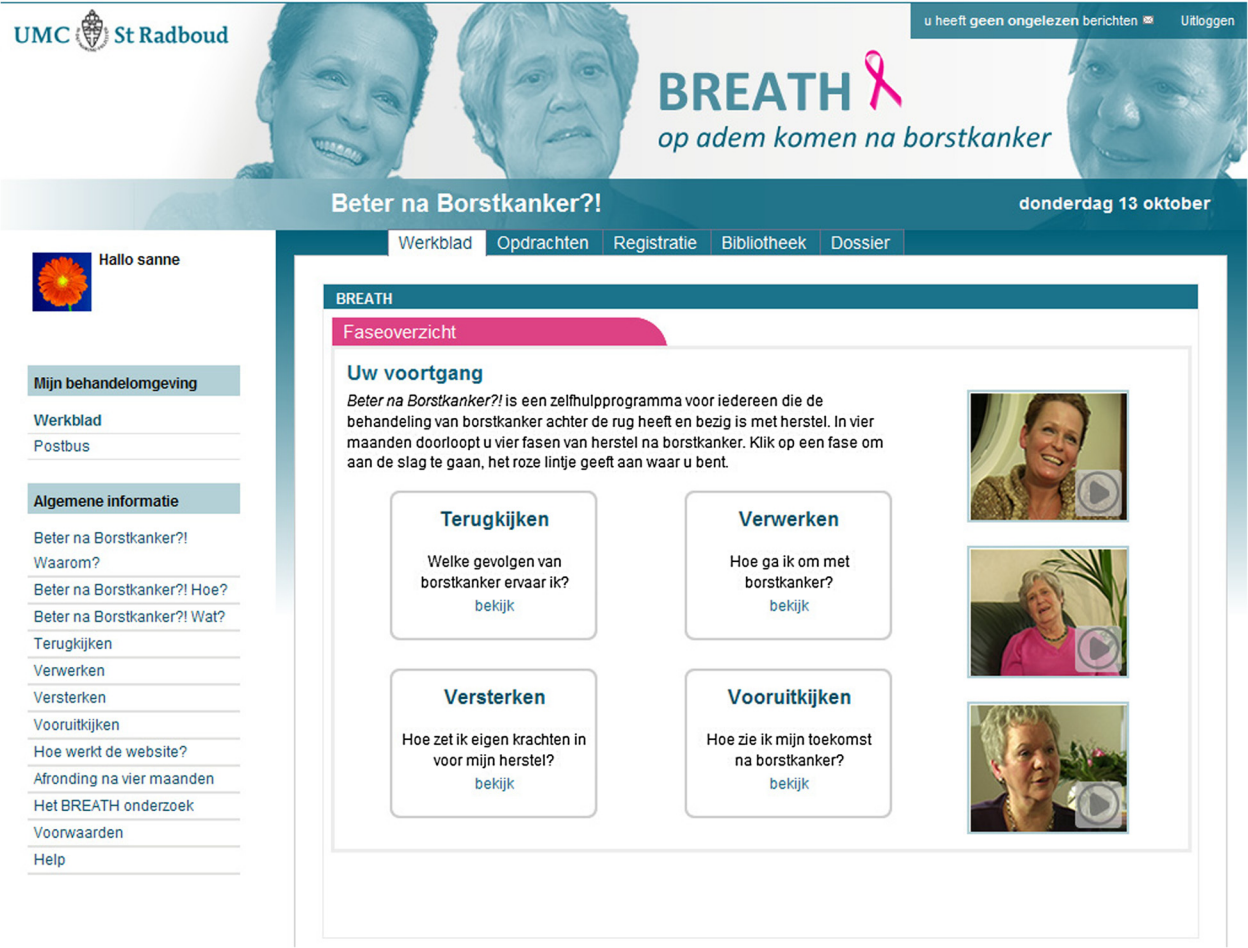
Screenshot of the BREATH intervention with four-month phase structure.

The non-guided Internet-based self-management BREATH intervention uses CBT techniques and guides BCS chronologically through the transition from ‘breast cancer patient’ to ‘survivor’. It is a preventive, early-intervention program that is available to all BCS and does not require screening. The protocol has a fixed structure that covers four months, representing four different phases of recovery after breast cancer: 1) Looking back [‘Terugkijken’], 2) Emotional Processing [‘Verwerken’], 3) Strengthening [Versterken’], and 4) Looking ahead [‘Vooruit kijken’]. These four phases are visually recognizable on the homepage of the intervention (see Figure 2). Each phase equals one month and has a fixed structure that covers four weeks, targeting consecutively psychoeducation, problems in everyday life, social environment, and empowerment. Universal re-entry topics for recovery after curative breast cancer treatment are organized within the fixed structure of the 16-week intervention (see Table 1 for some examples). At the start of the intervention, only the first week is available. During the course of the intervention, every week new information is unlocked and available to patients. The prescribed use of the intervention is one hour per week, which is a total exposure of 16 hours during the course of the four months of the intervention. However, no conditions are attached to the use or the time investment of the intervention. It is up to the BCS how and to what extent they use the intervention.

**Table 1:** Thematic content and structure of the BREATH intervention

**PHASE/MONTH**	**WEEK**			
	**Psychoeducation**	**Problems in everyday life**	**Social environment**	**Empowerment**
**1. Looking back**	· Getting started	· Emotional effects of breast cancer diagnosis and treatment	· Breast cancer within the family	· Personal strengths
	· Self-help contract	· Explanation of CBT	· Reactions of children	· Resources of strength
	· Personal intervention goals		· Reactions of partner	
**2. Emotional processing**	· Coping with life events	· Coping with breast cancer	· Social support	· Knowledge as power
	· Inventory of previous life events	· Personal grief	· Personal support network	· Coping with information
			· Sexuality	· Relaxation
**3. Strengthening**	· Physical consequences after breast cancer treatment	· Dealing with physical consequences	· Return to work	· Balancing strengths
		· Building up physical activity	· Talking about breast cancer at work	· Spirituality
**4. Looking ahead**	· Personal change after breast cancer	· Fear of recurrence	· Communication with care providers	· Future after breast cancer treatment
		· Coping with fear		
	· Recovery as priority		· Patient role less central	· Ahead on your own

#### Intervention functionalities

The most important functionalities of the BREATH intervention are the phase and week overview. Other functionalities include a library with background information, a personal notebook and a mailbox for technical assistance. Each week overview is filled with working ingredients surrounding a re-entry topic. Working ingredients include Information (25 scripts), Assignment (total 48 tasks), Assessment (total 10 tests) and Video (39 clips). Being a self-management program, the focus of the multi-modal intervention is on the information and the assignments. Assignments are for example writing tasks, social engagement or conversation tasks and aim to increase skill-building. Other elements of program interactivity included in the BREATH intervention are assessments or tests to be used by the patient as a screening instrument of potential problems. Tests are for example on topics concerning depressive mood after breast cancer treatment, fear of recurrence, and post-treatment fatigue. The tests are followed by automated feedback using a traffic light model (green-orange-red), with red illustrating elevated symptoms including the advice to contact a professional. The videos in the BREATH intervention are extracted clips from recorded interviews with three women who completed curative breast cancer treatment. The women in these peer modeling videos are of different ages and social backgrounds to increase recognition and empathy of the heterogeneous group of BCS.

#### Support

The BREATH intervention is fully-automated and non-guided and is delivered without professional support of a therapist. Human support is only available for technical assistance by the researcher. Through email, the researcher can be contacted and availability of this support is only during work-week and hours (Monday-Friday/9 am-6 pm). During the first login, a welcome page opens automatically with a demonstration video to secure basic knowledge of intervention functionality. The demonstration video stays available in the library of the intervention. Every week and on pre-specified times, standardized emails are sent to intervention users as a reminder that they have access to a new week of information. These support emails intend to reduce attrition by reminding users to return to and use the BREATH intervention [22].

#### Privacy

Intervention users are registered by the researcher and receive a unique user name and automated password (changeable later). Patients receive an invitation email with a statement of acceptance of conditions of use. The researcher has access to view the profile of the patient and the identity of the researcher is visible to the patient.

### Usual care

The control group of this RCT has access to usual care. The control condition reflects the natural course of recovery after curative breast cancer treatment. In the Netherlands, standard follow-up of breast cancer involves appointments every three months with a medical professional. The control group may freely use information about breast cancer on the Internet, but does not have access to the protected website with the BREATH intervention.

### Study outcome measures

Demographic characteristics will be gathered by self-report using questionnaires. Information on diagnosis will be obtained from the patient’s physician and medical record. Questionnaires are filled out online with RadQuest software (developed by the department of Medical Psychology, Radboud University Medical Centre, Nijmegen). Participants will receive an invitational email with a link to complete the questionnaires. For an elaborate overview of primary and secondary outcome measures, see Table 2.

#### Primary outcomes

*Psychological distress* will be assessed with the Symptom Checklist 90-items (SCL-90). The SCL-90 covers a broad range of psychological functioning from healthy persons to psychiatric patients and has good reliability, discriminant validity [34] and is sensitive to change through psychological intervention [35,36]. By using the SCL-90, minor changes in psychological functioning of less distressed BCS can be assessed.

*Psychological empowerment* will be measured with the Cancer Empowerment Questionnnaire (CEQ). The CEQ measures psychological empowerment as an outcome of empowerment processes in the individual patient [31]. The CEQ presumes that people with cancer can derive strength from themselves (intrapersonal subscale; Personal Strength), as well as from their social surroundings (interpersonal subscales; Social Support, Health Care, Community) [37].

#### Secondary outcomes

*Anxiety and depressive states* will be assessed with the Hospital Anxiety and Depression Scale (HADS) [38], which has shown good reliability and validity in oncological settings [39,40], Dutch medical patients [41], and BCS [42].

*Quality of life* related to breast cancer will be measured with the Dutch version of the European Organization for Research and Treatment of Cancer (EORTC) Quality of Life Questionnaire Core 30 (QLQ-C30) [43] and Breast Cancer Module (QLQ-BR23) [44]. Both questionnaires have demonstrated good psychometric properties in BCS [45,46].

*General distress* will be measured with the Dutch version of the Distress Thermometer (DT) [47]. The one-item screening tool (thermometer) has proven good sensitivity and specificity in breast cancer patients [48,49].

*Illness perceptions* will be assessed with the Illness Cognitions Questionnaire (ICQ) [50], which has good psychometric properties in patients with chronic medical conditions [50,51].

*Remoralization* refers to the restoration of morale (in this study after the completion of breast cancer treatment) and will be measured with the Remoralization Scale (RS) [52].

*Personal control* over life in general, or sense of mastery, will be measured with the Mastery Scale [53], which has shown to be a predictor of psychological adjustment in the year following a breast cancer diagnosis [13,54].

*Positive adjustment* following breast cancer will be measured with the Positive Adjustment Questionnaire (PAQ) [55].

*Coping* with the experience of breast cancer will be measured with the Dutch version of the Impact of Event Scale (IES) [56-58] and the Brief COPE [59,60].

*Self-efficacy* with regard to complaints (in this study as a result of breast cancer) will be measured with the Self-Efficacy Scale (SES), which has previously been used to assess self-efficacy concerning post-cancer fatigue [61,62].

*Fear of cancer recurrence* and the impact of cancer worries on daily life will be measured with the Dutch extended version [63,64] of the Cancer Worry Scale (CWS) [65,66] and the Cancer Acceptance Scale (CAS) [64,67].

*Fatigue* will be measured with the fatigue severity subscale of the Checklist Individual Strength (CIS-fatigue), which has good psychometric properties in cancer survivors [36].

*Family communication about breast cancer* will be measured with a modified version of the Openness to Discuss Hereditary Cancer in the Family (ODHCF) scale [68,69]. For this study, the ODHCF was adapted for women who completed curative breast cancer treatment.

*Personality* factors will be measured with the Dutch version [70] of the Big Five Inventory (BFI) [71].

The costs of *health care utilization* will be collected through a modified version of the Trimbos/iMTA questionnaire for Costs associated with Psychiatric illness (TiC-P) [72]. Questions about the use of breast cancer-specific medication and care are added to the first part of the TiC-P concerning direct costs of mental health care and medicine utilization.

#### Process outcomes

For the BCS in the experimental group, technical data on the use of the BREATH intervention will be collected in addition to the standardized questionnaires. Because the actual use of this newly developed intervention in everyday life is unclear, usage variables are recorded to reflect the actual exposure to the intervention content. Frequency and duration of logins, website activity, and other significant usage statistics will be evaluated [73].

#### Other outcomes

For all patients, medical disease-specific data will be provided by the hospital where the patient is recruited, or will be collected from the (electronic) medical record by the researcher. Also, in the post-treatment (T1) measure, a question on breast-cancer specific Internet use will be listed.

### Sample size calculation

Based on the two primary outcomes of the BREATH study, effect on patient level is defined as a decrease in psychological distress (as measured with the SCL-90) *or* an increase in empowerment (as measured with the CEQ). Therefore, effectiveness of the BREATH intervention is demonstrated when one of the two effects is significant. Sample size calculation is based on the SCL-90, since the SCL-90 has proven sensitive to change through psychological face-to-face intervention in fatigued cancer survivors [36] and information on sensitivity to change of the CEQ is unknown. Significance level of the sample size calculation was adjusted to p ≤ 0.25 to keep the overall chance for type-I errors on 5%. To detect a significant difference (0.25) with 80% power between intervention and control group on the SCL-90, a sample size of 128 BCS (64 in each group) is needed. This sample size calculation is based on a medium effect size of 0.50 of the BREATH intervention, which has proven to be a high effect size for non-guided psychological interventions [74]. Based on a systematic review on attrition in randomised controlled trials of Internet interventions for anxiety and depression [75], we take into account a 25% study drop-out rate. This results in a maximal sample size of 170 BCS that need to be included in the BREATH trial in case of considerable drop-out.

**Table 2:** Primary and secondary outcome measures of the BREATH study

**Questionnaires**		**Response format**	**Example questions**	**Timepoints**
**Primary outcome measures**				
Psychological distress	Symptom Checklist 90 (90 items)	5 point Likert scale	During the past 7 days about how much were you distressed or bothered by:	T0; T1; T2; T3
				
	· Anxiety (10 items)	* range 90–450	*Feeling fearful (anx)	
	· Agoraphobia (7 items)			
	· Depression (16 items)		* Feelings of worthlessness (depr)	
	· Somatisation (12 items)		* Numbness or tingling in parts of your body (som)	
	· Obsessive-compulsive behaviour (9 items)			
	· Interpersonal sensitivity (18 items)		* Feeling that people are unfriendly of dislike you (int.sens)	
	· Hostility (6 items)		* Nervousness or shakiness inside (anx)	
	· Sleep (3 items)			
Psychological empowerment	Cancer Empowerment Questionnaire (40 items)	5 point Likert scale		T0; T1; T2; T3
	· Personal strength (19 items)	* range 40-200	* I know what I am good at (pers.str)	
	· Social support (9 items)		* The people around me take me for who I am (soc.sup)	
	· Community (6 items)		* In our society people with breast cancer are considered wholly (com)	
	· Health care (6 items)		* My health care professionals are there when I need them (h.care)	
**Secondary outcome measures**				
Anxiety and depressive states	Hospital Anxiety and Depression Scale (14 items)	4 point Likert scale	During the past week:	T0; T1; T2; T3
				
	· Anxiety (7 items)	* range 0-21 (subscales)	* I am restless and can’t keep still (anx)	
	· Depression (7 items)	* range 0-42 (total)	* I feel miserable and sad (depr)	
Breast cancer- related quality of life	EORTC-C30 (30 items)	4 point Likert scale	During the past week:	T0; T1; T2; T3
				
	· Functional scales (physical, role, emotional, social, and cognitive functioning - 15 items)	* range 15-60	* Has your physical condition interfered with your family life? (role.func)	
	· Symptom scales (fatigue, pain, nausea/vomiting - 7 items)	* range 7-28	* Have you had difficulties remembering things? (cog.func)	
	· Single symptom items (6 items)	* range 6-24 7-point linear analogue scale; range 2-14	* Did pain interfere with your daily activities? (pain)	
	· Global health and global quality of life (2 items)		* How would you rate your overall quality of life during the past week?	
	EORTC-BR23 (23 items)	4 point Likert scale	During the past week:	T0; T1; T2; T3
				
	· Functional scales (body image, sexual functioning, sexual enjoyment, future perspective - 8 items)	* range 8-32	* Did you find it difficult to look at yourself naked? (body.im)	
	· Symptom scales (arm symptoms, breast symptoms, systemic therapy side effects, upset by hair loss - 15 items)	* range 15-60	* Did you have a swollen arm or hand? (arm.symp) During the past 4 weeks:	
			* To what extent were you interested in sex? (sex.enj)	
General distress	Distress Thermometer (1 item)	11 point Likert scale		T0; T1
		* range 0-10	* Circle the number that best describes how much distress you have been experiencing in the past week including today.	
Remoralization	Remoralization Scale (12 items)	4 point Likert scale		T0; T1
		* range 12-48	* I am in control of my life	
			* I take a positive attitude toward myself	
Personal control	Mastery scale (7 items)	5 point Likert scale		T0; T1; T2; T3
		* range 7-35	* What happens to me in the future mostly depends on me	
Positive adjustment to cancer	Positive Adjustment Questionnaire (39 items)	1-7 scale	Since you found out about your illness:	T0; T1; T2; T3
				
	· Fulfilment (9 items)	* range 9-63	* My life is not limited by my illness (ful)	
	· Re-evaluation (9 items)	* range 9-63	*I see life differently (re-ev)	
	· New ways of living (10 items)	* range 10-70	* I can take things more in my stride (new.w)	
	· Valuing life (7 items)	* range 7-49	* I value life a lot more now (val)	
Coping with the experience of cancer	Impact of Event Scale	4 point Likert scale		T0; T1
	· Intrusion (7 items)	* range 13-52	* I had dreams about it (intr)	
		· Avoidance (8 items)	* I tried not to think about it (avoid)	
			* I tried not to talk about it (avoid)	
	Brief COPE (28 items)	4 point Likert scale		T0; T1
	· Active coping (2 items)	* range 0-6 (subscales)	* I’ve been taking action to try to make the situation better (act.cop)	
	· Planning (2 items)		* I’ve been learning to live with it (acc)	
	· Positive reframing (2 items)		* I’ve been giving up to attempt to cope (behav.dis)	
	· Acceptance (2 items)			
	· Humor (2 items)			
	· Religion (2 items)			
	· Using emotional support (2 items)			
	· Using instrumental support (2 items)			
	· Self-distraction (2 items)			
	· Denial (2 items)			
	· Venting (2 items)			
	· Substance use (2 items)			
	· Behavioral disengagement			
			* I’ve been criticizing myself (self-bl)	
	· (2 items)			
	· Self-blame (2 items)			
Self-efficacy	Self Efficacy Scale (7 items)	4 point Likert scale		T0; T1
		* range 7-28	* Whatever I do, I cannot change my complaints	
			* I think I could positively influence my complaints	
Fear of cancer recurrence	Cancer Worry Scale (8 items)	4 point Likert scale	During the past month:	T0; T1; T2; T3
				
				
		* range 8-32	* How often have you thought about your chance of getting cancer (again)?	
			* Have these thoughts affected your mood?	
	Cancer Acceptance Scale (2 items)	4 point Likert scale		T0; T1
		* range 2-8	* I worry about the cancer returning	
			* I am anxious about my health	
Fatigue Severity	Checklist Individual Strength	7-point Likert scale		T0; T1; T2; T3
	· subscale Fatigue Severity (8 items)	* range 8-56	* I feel tired	
			* I am rested	
			* Physically I feel exhausted	
Family communication	ODHCF (14 items)	5 point Likert scale		T0; T1; T2; T3
	· Nuclear family (7 items)	* range 7-35 (subscales) (or less if not applicable; no partner/ children/ parents/ siblings)	* My partner doesn’t (nucl.fam) / parents don’t (fam.or) want me to talk about breast cancer	
	· Family of origin (7 items)		* My children (nucl.fam) / siblings (fam.or) often don’t know what to say or do, when I’m feeling down because of breast cancer.	
Illness perceptions	Illness Cognition Questionnaire (18 items)	4 point Likert scale		
	· Helplessness (6 items)	* range 6-24 (subscales)	* My illness controls my life (help)	T0; T1; T2; T3
	· Acceptance (6 items)		* I can accept my illness well (acc)	
	· Perceived benefits (6 items)		* I have learned a great deal from my illness (perc.ben)	
Personality	Big Five Inventory (44 items)	5 point Likert scale	I see myself as someone who:	T0; T1
	· Neuroticism (8 items)	* range 1-5 (subscales)	* Worries a lot (neu)	
	· Extraversion (8 items)		* Is full of energy (ext)	
	· Openness to experience (10 items)		* Tends to be disorganized (con)	
	· Conscientiousness (9 items)		* Is considerate and kind to almost everyone (agr)	
	· Agreeableness (9 items)			
Health care utilization	Trimbos/iMTA questionnaire for Costs associated with Psychiatric illness (TiC-P)		Since completion of primary curative breast cancer treatment (T0) / In the past four months (T1; T3) / In the past two months (T2):	T0; T1; T2; T3
	· Contacts with medical or mental health care professionals (14 items)	Yes/no; frequency	* Did you visit a psychologist? (con)	
	· Hospitalization (1 item)	Yes/no; reason Yes/no; dose and frequency	* Did you use antidepressants? (med)	
	· Use of breast cancer- specific, psychiatric and other medication (8 items)	Yes/no; costs	* Did you take part in a breast cancer rehabilitation program? (other)	
	· Other costs (10 items)			

### Statistical analysis

A general linear model for repeated measurements (by the method of mixed linear model) will be used to analyze the effect of the BREATH intervention on the two primary outcome variables (distress and empowerment). Chi square (categorical variables), ANOVA (normally distributed continuous variables), and Kruskall-Wallis (non-parametric variables) will be used to asses baseline characteristics between groups. Analysis will be done according to intention-to-treat methodology.

### Time line of the BREATH study

Recruitment of participants began in 2010. Primary end-points (baseline T0 and post-intervention T1) and all follow-up measures (T2-T3) are expected to be completed in January 2013.

### Ethical issues

The BREATH study has been approved by the Medical Ethics Committee (CMO) of the Radboud University Medical Centre, Nijmegen in the Netherlands (CMO protocol number 2009/144) [see additional file 1]. The study has also been approved an registered by the local ethical committees of each hospital setting. Registration number of the Netherlands Trial Register is NTR2935.

## Discussion

This study will evaluate the effectiveness of a non-guided Internet-based self-management intervention for BCS to decrease psychological distress and increase empowerment. To the best of our knowledge, this is the first online self-management intervention specially designed for the BCS after completion of primary breast cancer treatment. Using the Internet, the BREATH intervention provides a novel and easy-accessible approach to reduce at an early stage the impact of psychological problems that may arise after the completion of medical treatment. In the long term this study may contribute to early prevention of psychological problems in BCS. The BREATH study is innovative in the field of psycho-oncological intervention research. The results of this study will provide novel insights in whether common-used CBT-techniques can foster patients’ own strengths towards adjustment to breast cancer. Also, whether it is possible to address both distressed and non-distressed BCS with the same intervention and without guidance of a psychologist, or other health care professional.

When effective, the BREATH intervention will be implemented in follow-up care of BCS. However, in light of the results of the RCT and experiences of the participants it is possible that the content, dose or structure of the intervention will have to be adjusted. For conducting high quality research, we have chosen to offer the BREATH intervention in a structured way with new information being disclosed every week. This trial will provide information on whether BCS prefer a more flexible approach and access to the entire intervention content. Also, the results of this study will have to show whether three months after completion of primary breast cancer treatment is the right time point to start with online self-management. Last, the results of this multicentre RCT will provide insights in who benefits from BREATH, or which subgroups of BCS gain most effect of the intervention. Information on subgroups might lead to personalizing psychosocial cancer treatment in future survivorship care of breast cancer survivors. The BREATH intervention provides a minimal intervention that can fill the gap between the needs and availability of psychosocial support after breast cancer treatment.

## Abbreviations

BCS: Breast cancer survivors; CBT: Cognitive behavioural therapy; CEQ: Cancer Empowerment Questionnaire; CMO: Medical Ethics Committee; RCT: Randomised controlled trial.

## Competing interests

The authors declare that they have no competing interests.

## Authors’ contributions

SvdB is PhD student and responsible for patient recruitment, data collection, data analysis, and drafting this manuscript. JP and PO developed the original idea. JP is project leader and supervises the trial. JP and SvdB designed the intervention and wrote the intervention content. PO is co-grant applicator and takes a place on the advisory board, together with MG. MG also contributed to the content of the intervention. All authors read and approved the final manuscript.

## Pre-publication history

The pre-publication history for this paper can be accessed here:

http://www.biomedcentral.com/1471-2407/12/394/prepub
